# Effect of type and concentration of hydrocolloids on the rheology, water mobility, and 3D printing properties of pitaya fruit-based ink

**DOI:** 10.3389/fnut.2025.1668661

**Published:** 2026-01-06

**Authors:** Jacob Ojobi Omedi, Cheng Chen, Angelo Uriho, Shuning Zhang, Li Liang, Qibo Zou, Yan Xu, Min Zhang, Yuchuan Wang, Weining Huang

**Affiliations:** 1 State Key Laboratory of Food Science and Resources, and School of Food Science and Technology, Jiangnan University, Wuxi, China; 2 Fortune Bakery Co., Ltd., DaoXianCun Group, Zhangjiagang, China; 3 Key Laboratory of Industrial Biotechnology of Ministry of Education, School of Biotechnology, Jiangnan University, Wuxi, China

**Keywords:** 3D printing, hydrocolloids, pitaya fruit, rheological properties, textural profile, water mobility

## Abstract

In this study, the effects of three hydrocolloids, xanthan gum (XG), arabica gum (AG) and carboxymethylcellulose (CMC), at two concentrations on the rheological, water mobility and 3D printing characteristics of pitaya fruit-based inks were investigated. The results showed that hydrocolloids restricted water mobility by increasing the immobilized water (T_22_) with higher content in CMC (97.5%−98.01%), AG (96.28%−97.26%), and XG (95.35%−95.38%), at 9 g/100 g than 6 g/100 g hydrocolloid concentration in the hydrocolloid-fruit inks. The rheological properties, based on the apparent viscosity, G′ and G^′′^ were increased in the pitaya fruit inks, especially in presence of CMC, then AG and XG at higher than lower concentration. Moreover, the printing precision improved, while the textural profile based on hardness and resilience increased, adhesiveness and springiness decreased, and influenced the color profiles in the hydrocolloid-fruit ink printed objects containing XG, then CMC and AG at 9 g/100 g than 6 g/100 g concentration. These changes were attributed to the increased water holding capacity in presence of different hydrocolloids at higher concentrations which reduced water mobility, slow water molecules re-orientation, and increased pseudoplastic and solid-like (G′ higher than G^′′^) character in the hydrocolloid-fruit inks: this could have led to the better retention of the 3D printed structure. The results presented demonstrated the influence of different types of hydrocolloids at varying concentrations on altering the physicochemical and technological properties of high moisture content fruit substrate-based inks for 3D food printing applications. In addition, these findings pave the way for further studies on the development of natural and health-promoting fruit substrate-based 3D printed snack food products.

## 1 Introduction

The increased consumer demand for healthy diets rich in nutritional and functional ingredients has continued to guide several food research and industry efforts. Moreover, the intake of fruits as a common part of the human balanced diet are rich sources of micronutrients such as vitamins and minerals, antioxidants and other bioactive compounds (1–3). One such fruit is pitaya, natively grown in Central and South America, has increasingly been cultivated in other parts of the world, has attracted global interest for use in several product development due to its exotic appearance, rich nutritional value, and health properties (4–7). However, in individuals with chewing difficulties (e.g., elderly), the consumption of fresh fruits may be considered too stiff to chew in their raw forms, while when processed they tend to lose some of their nutritional and functional attributes (8). To improve the texture of the fruit for safe swallowing with minimal effect on the nutritional and functional attributes, the use of texture modifying ingredients such as hydrocolloids and emerging technologies such as 3D food printing, respectively, may be useful (9).

The use of 3D printing technology in food offers diverse possibilities for new innovative food products (10). As an emerging technology, 3D printing in food could potentially revolutionize the eating habits of consumers, including the individuals with chewing difficulties (10). In published studies, several food-based materials, including those based on gels (11–13) and dough (14–17), have been used in the 3D printing process. Interestingly, even though a fruit substrate in dough system was successfully used as a food ink during 3D printing (16), there are fewer known and published studies on the use of entire fruit-based substrates in 3D printing. This was attributed to the very high-water content and low viscosity of fruit substrate inks which greatly reduces their plasticity and molding characteristics, making them too fluid to be used for effective 3D printing into edible inks (18). To mitigate this shortcoming, the use of different functional ingredients such as hydrocolloids and starches has been proposed (19, 20).

Hydrocolloids are diverse long chain polymers generally characterized by their ability to form viscous gels and/or dispersions in water (21), a property that may be useful in the enhancement of the printability of high-water containing materials such as fruits during 3D printing process. For instance, in a 3D printed pork paste, the presence of xanthan or guar gum increased the viscosity levels through improved association between the glucose backbone and trisaccharide side chains, and increased crosslink between water molecules and the galactose side chains, respectively (18). The potential interactions such as covalent bonding, electrostatic bonding and hydrophobic hydrogen bonding between hydrocolloids and fruit-based materials may significantly improve the printing performance of fruit-based inks (18). These improvements may be associated with the ability of the hydrocolloids to alter the water distribution and mobility patterns (22, 23), invariably influencing the rheological properties of the resulted printed object. In addition, the influence of the hydrocolloid type and the concentration used on the effectiveness of 3D printed fruit-based inks is still scare and limited (24, 25). Therefore, the careful selection of the type of hydrocolloid and the appropriate concentration could be vital to ensure improved rheological properties, water holding capacity of the ink and enhanced textural profiles of the 3D printed fruit-based object.

Therefore, the aim of this study was to investigate the suitability of 3 hydrocolloid types, namely, xanthan gum (XG), arabica gum (AG), and carboxymethylcellulose (CMC), on the printability of red pitaya fruit puree as a fruit-based ink for 3D printing. To achieve this objective, we evaluated the effect of six hydrocolloid concentration (0.5, 1, 2, 3, 6, 9 g/100 g) on the technological properties (water holding capacity, flow properties) of pitaya fruit puree. At the selected optimal concentration (s), the effect of hydrocolloid type and concentration on the physicochemical properties (pH, moisture content), rheological properties (storage modulus (G′), loss modulus (G^′′^), apparent viscosity) and water mobility of the hydrocolloid-fruit ink, and its impact on the color profile, textural profile and printing stability of the 3D printed hydrocolloid-fruit inks were investigated.

## 2 Materials and methods

### 2.1 Materials

Fresh red pitaya fruit (*Hylocereus undatus*) was purchased at commercial maturity (14 ± 1 °Brix) from a local supermarket in Wuxi (China). Hydrocolloids of xanthan gum (XG) (GB 1,886.41) (Xinjiang Meihua Amino acid Co., Ltd, Xinjian, China), Arabica gum (AG) (GB 29,949–2013) (Dingli Biodegradable Materials Co., Ltd, Shandong, China), and carboxymethylcellulose (CMC) (GB 1,886.232) (Shanghai Changguang Enterprise Development Co., Ltd, Shanghai, China) were purchased and used.

### 2.2 Preparation of the hydrocolloid-fruit inks

Pitaya fruits were washed to remove dust and dirt, then peeled to remove its peel from the flesh of the fruit. The flesh of the pitaya fruit was the crushed and homogenized in a food processor, followed by passing through a standard mesh (particle size, 100 μm) to remove the seeds and produce a uniform consistent pulp. To investigate the influence of the type and concentration of hydrocolloids on the properties of pitaya-based fruit inks, three hydrocolloids, including, XG, AG, and CMC, were singly added to pitaya fruit pulp at concentrations of 0.5, 1, 2, 3, 6, and 9 g per 100 g of pitaya fruit pulp and the thoroughly mixed (stirred continuously for 30 min) until a homogeneous mixture was obtained. To prevent lump formation and promote uniform hydrocolloid dispersion, a small amount of pitaya fruit pulp was initially mixed with the calculated amount of the hydrocolloid to form a paste, followed by adding the remaining pulp. The hydrocolloid-fruit inks were stored overnight in a refrigerator (4 °C) to allow hydration. A portion was used to analyze and select the optimal concentration for use in the subsequent experiments.

### 2.3 Physicochemical and technological properties of the selected hydrocolloid-fruit inks

#### 2.3.1 Flow properties of the hydrocolloid-fruit inks

The flow properties of the hydrocolloid-fruit inks were determined using the NDJ-5S digital rotational viscometer (Nanbei Instrument Ltd, Jinshui, Zhengzhou, China) as described by (26) with some modifications. The instrument was designed with 5 cylindrical rotors (0#, 1#, 2#, 3#, 4#) and 4 different velocities (6, 12, 30, and 60) rpm. In this study, rotor 3# was used and the machine set at automatic switch to enable free selection of proper rotating speed to measure the viscosity in the range 2,000–20,000 mPa.s in the hydrocolloid-fruit ink samples. Each measurement was performed in triplicate.

#### 2.3.2 Water hydration capacity (WHC) of the hydrocolloid-fruit inks

WHC of the samples was determined following the method described by (27) with some modifications. Briefly, 0.2 g of the samples were weighed and dispersed in 30 mL of deionized water in centrifuge tubes. The dispersions were hydrated for 1 h in a water bath at 60 °C, and then placed in cold water for 10 min. The samples were centrifuged at 10,000 × *g* for 15 min. The WHC was calculated using the following equation.


$$\begin{array}{l} \text{WHC (g/g)}=\left[\left({\text{W}}_{T+R}-{\text{W}}_{T}-{\text{W}}_{P}\right)/{\text{W}}_{P}\right]. \end{array}$$

Where W_*P*_ is the weight of the sample, W_*T*+*R*_ is the weight of the centrifuge tubes and the residue after the supernatant was removed, and W_*T*_ is the weight of the empty centrifuge tube.

#### 2.3.3 Moisture content of the selected hydrocolloid-fruit inks

Moisture content of the selected hydrocolloid-fruit inks was determined by the gravimetric method using a one stage drying process (130 °C for 2 h) as described by (28). Three measurements were performed for each sample. The moisture content was expressed as grams of water over grams of total weight (g/100 g).

#### 2.3.4 pH of the selected hydrocolloid-fruit inks

The pH of the selected hydrocolloid-fruit inks was determined. Briefly, 10 g of sample was homogenized with 90 mL of distilled water. The pH was recorded using a pH meter (Mettler Toledo, China).

### 2.4 Determination of the dynamic oscillatory properties of the selected hydrocolloid-fruit inks

The dynamic oscillatory properties of the hydrocolloid-fruit inks were determined using a rheometer (DHR-3, Waters Corporation, USA) using a 25 mm diameter sandblasted parallel plate system with a gap of 1,000 μm. Before the analysis, the hydrocolloid-fruit inks were equilibrated at 25 °C for 4 min. The apparent viscosity was measured with shear rates ranging from 0.1 to 100 1/s. The Oscillation strain was performed at a frequency of 1 Hz within the range of 0.01%−100%. The dynamic viscoelasticity measurements were performed by applying a constant strain of 1.01% at 25 °C with an angular frequency sweep test from 0.1 to 100 rad/s, and the storage modulus (G′) and loss modulus (G^′′^) were measured. All samples loaded in the rheometer were allowed to rest for 10 min before starting the measurement. To prevent the sample from drying during the long measuring time, the exposed surfaces around the samples were coated with corn oil. All determinations were performed at 25 °C and three replications were performed.

### 2.5 Water mobility analysis using low field nuclear magnetic resonance (LF-NMR) spectrometer

The water mobility of the selected hydrocolloid-fruit inks was determined using LF-NMR spectrometer (MesoMR23-060 V-I; Niumag Electric Corporation, China) according to method described by (29) with some modifications. Briefly, 4 g of sample wrapped in the polyethylene bag was transferred into the NMR probe (25 mm external diameter). The transverse relaxation time (T_2_) were measured using a Carr–Purcell–Meiboom–Gill (CPMG) pulse sequence. The pulse parameters were: waiting time (TW) = 1,000 ms, two peaks interval (TE) = 0.4 ms, number of points of determination (NECH) = 6,000, interval between points (SW) = 100 KHz, pre-amplifier gain (PRG) = 2, number of scans (NS) = 6. Each measurement was performed in triplicate.

### 2.6 3D printing of the hydrocolloid-fruit inks

A FOODBOT-MF 3D food printer (Hangzhou Shiyin Technology Co., Ltd., Zhejiang, China) as described by (30) was used for the 3D printing process. A 3D design (.stl file format) with a cube shaped model (20 mm × 20 mm × 12 mm) was created using 3D printing slicing software (version 4.0.1; Cincinnati, OH, USA). The selected hydrocolloid-fruit inks were loaded in a syringe equipped with a 0.84 mm nozzle diameter. All the printing experiments were carried out at 25 °C, and the printed products were deposited on a plastic (polyvinyl chloride) bed and analyzed.

### 2.7 Determination of dimensional printing deviation

The printing performance of the different hydrocolloid-fruit inks were measured for dimensional printing deviations relative to the selected cube shaped model at 3 dimensions (Length, width, height) as described by Qiu et al. (30) with some modifications.

### 2.8 Textural properties of printed hydrocolloid-fruit inks

The texture characteristics of the printed objects were measured using a textural analyser (TA-XT plus, Stable Micro System Company, UK) following the method described by Qiu et al. (30) with some modifications. For texture analysis, a cylindrical model (10 mm × 10 mm × 10 mm) was used to print the hydrocolloid-fruit inks. Prior to testing, the analyzer was calibrated for the weight and height, then the printed samples were put in the center of the platform and a cylinder probe (diameter = 35 mm) was applied for the determination of force-time curves. The test conditions used were, pre-test speed: 2 mm/sec, test speed: 2 mm/sec, post-test speed: 2 mm/sec, compression strain: 40%, at ambient temperature (25 °C).

### 2.9 Color analysis of printed hydrocolloid-fruit inks

For color analysis, a cylindrical model (10 mm × 10 mm × 10 mm) was used to print the hydrocolloid-fruit inks. The color of the printed inks was determined using a precision colorimeter (Konica Minolta CR-400, Japan) as described by (22). The results were reported as L^*^ (lightness, white black), a^*^ color-opponent dimension, red-green), and b^*^ (color-opponent dimension, yellow-blue).

### 2.10 Statistical analysis

Results of three independent assays were presented as mean values. Data was compared using one-way analysis of variance (ANOVA), while multiple comparisons of data was performed by Duncan's test at *p* < 0.05 level of significance using SPSS 26.0 (SPSS Inc., Chicago, IL, USA). A Pearson correlation test was performed to explore the effect of the type of hydrocolloid used and its concentration on the parameters of the hydrocolloid-fruit inks and the printed objects.

## 3 Results and discussion

### 3.1 Technological properties for the selected hydrocolloid-fruit ink

In the 3D printing process, the homogeneity and appropriate flow properties of the 3D printing ink material are vital to support its structure during and after the 3D printing process (31). To evaluate the optimal concentration of the hydrocolloid in the hydrocolloid-fruit ink for 3D printing, the water holding capacity (WHC) and flow properties were determined at increasing hydrocolloid concentration in the pitaya fruit. The results showed that the WHC were enhanced at higher hydrocolloid concentration in the hydrocolloid-fruit inks (Supplementary Figure S1A). Subsequently, the highest WHC was shown in the hydrocolloid-fruit inks containing 9 g/100 g than 6 g/100 g hydrocolloid concentration. Moreover, at the same hydrocolloid concentration in the fruit inks, the WHC was highest in the presence of CMC (9 g/100 g: 0.223 g/g, 6 g/100 g: 0.129 g/g), then XG (9 g/100 g: 0.181 g/g, 6 g/100 g: 0.094 g/g) and least in AG (9 g/100 g: 0.060g/g, 6 g/100 g: 0.052 g/g) (Supplementary Figure S1A). This suggested that the type of hydrocolloid and the concentration influenced the WHC characteristics in the hydrocolloid-fruit inks (32). Similar to WHC values, the viscosity was highest in all inks prepared with hydrocolloid concentrations at 6 g/100 g and 9 g/100 g (Supplementary Figure S1B). At the observed values (Supplementary Figure S1B), the flow properties of the hydrocolloid-fruit inks may be at their optimum and able to support the structure of the resulted 3D printed material (31). Therefore, 6 g/100 g and 9 g/100 g concentration of the 3 hydrocolloids in the hydrocolloid-fruit ink was selected for the subsequent experimentation.

### 3.2 The pH, moisture content (MC) and water holding capacity (WHC) of the hydrocolloid-fruit inks

Despite pitaya' popularity, its use as an ink for 3D printing ink faces several drawbacks due to its high moisture content that makes its unsuitable for 3D printing (33–38). The changes in the pH, MC, and WHC of the selected hydrocolloid-fruit inks were presented in Table 1. The pH ranged from 5.56 to 6.51, with lower and higher values observed in XG and AG, and CMC, respectively, containing hydrocolloid-fruit inks. On the other hand, the MC and WHC was in the range of 74.95%−81.40%, and 0.05–0.22 g/g, respectively, in the hydrocolloid-fruit inks (Table 1). These results showed that the WHC increased and MC decreased at 9 g/100 g than 6 g/100 g of hydrocolloid in the hydrocolloid-fruit inks. Significantly higher WHC and lower MC was seen in inks prepared with CMC, then XG and least in AG. The decreased MC at higher concentrations of hydrocolloids was attributed to ability of the hydrocolloids to increase the WHC of the available water in the hydrocolloid-fruit inks (32). In addition, the source of the hydrocolloids, such as chemically modified (CMC), microbial (XG) and plant (AG), could have influenced the physicochemical and technological properties through enhanced molecular interactions which stabilized the hydrocolloid-fruit inks (32). This was confirmed in several studies where the addition of hydrocolloids and other water binding ingredients increased the WHC of the edible inks for the 3D printing process (39, 40). In this study, the changes in the pH, MC and WHC at 9 g/100 g than 6 g/100 g hydrocolloid concentration in the hydrocolloid-fruit inks may be vital for improved printing performance of pitaya fruit-based inks for 3D printing.

**Table 1:** Physicochemical and technological properties of the selected hydrocolloid-pitaya fruit mixtures.

**Sample**	**Parameter (s)**		
	**pH**	**MC (%)**	**WHC (g/g)**
Arabica-6 g/100 g	5.78 ± 0.01c	79.14 ± 1.48ab	0.052 ± 0.022a
Arabica-9 g/100 g	5.62 ± 0.01b	78.30 ± 2.17ab	0.06 ± 0.01a
Xanthan-6 g/100 g	5.56 ± 0.01a	81.40 ± 0.77b	0.094 ± 0.018ab
Xanthan-9 g/100 g	5.78 ± 0.01c	76.87 ± 3.00ab	0.181 ± 0.034c
CMC-6 g/100 g	6.34 ± 0.03d	78.96 ± 1.35ab	0.129 ± 0.010b
CMC-9 g/100 g	6.51 ± 0.01e	74.95 ± 2.15a	0.223 ± 0.001c

Mean (n = 3) with different lower-case letter in each column denotes difference (*p* < 0.05).

MC, Moisture content; WHC, Water holding capacity; CMC, Carboxymethylcellulose.

### 3.3 Effect of type and concentration of hydrocolloid on the dynamic oscillatory properties in hydrocolloid-fruit inks

**Figure 1:**
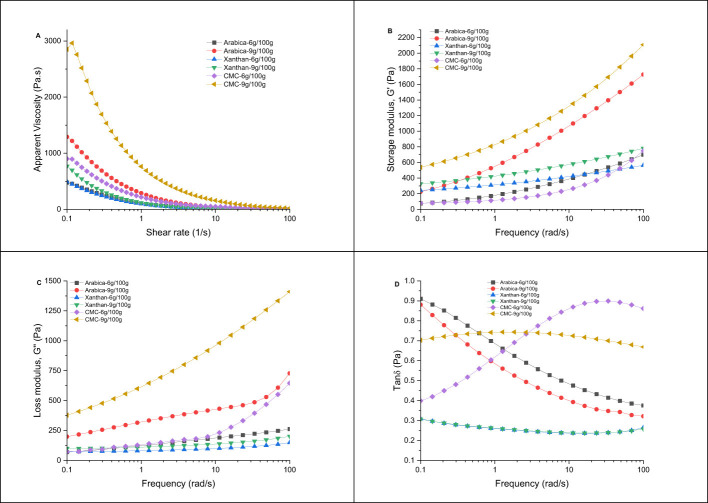
The apparent viscosity **(A)**, storage modulus **(B)**, loss modulus **(C)**, and tan δ **(D)** of the selected hydrocolloid-pitaya fruit ink samples.

The dynamic oscillatory properties of the edible inks significantly influence their ability to self-support during and after the 3D printing process (41). The dynamic oscillatory properties of the hydrocolloid-fruit inks were presented in Figure 1. As shown in Figure 1A, the apparent viscosity results showed that an increase in the shear rates led to the reduction in the viscosity of all the samples. This suggested that all the hydrocolloid-fruit inks exhibited a shear-thinning pseudoplastic character, a property desirable for the smooth extrusion of the food inks during the 3D printing process (30). The pseudoplastic character was attributed to the deterioration of the network structure with increased shear rate in the hydrocolloid-fruit inks (42). Moreover, higher apparent viscosities were observed in hydrocolloid-fruit inks prepared with CMC, then AG and least in XG, especially at 9 g/100 g than 6 g/100 g hydrocolloid concentrations at low shear rates (< 10 1/s) (Figure 1A). These observations were in agreement with the findings in a study where higher apparent viscosities were found in yam starch-based hydrogel inks prepared with guar gum, arabica gum, and chitosan than in the presence of xanthan gum (22). This suggested that the conformation formed in the different hydrocolloid-fruit inks could have been influenced by the type of hydrocolloid used in this study (32). In this study, the hydrocolloids used were plant-based (AG), chemically modified plant-based (CMC) and microbial-based (XG), each with distinct chemical structures and properties which may influence the rheological properties of the ink uniquely (32). For instance, in the hydrocolloid-fruit inks prepared with CMC and AG, the conformations formed might be less rigid, whereas a more rigid rod-like conformations might have been formed in XG containing inks which could easily orient during shear stress leading to significant shear thinning in the hydrocolloid-fruit inks (32, 43). Furthermore, at higher hydrocolloid concentration, more networks were formed between the hydrocolloids and fruit components through interactions such hydrogen bonds (32). Subsequently, the intermolecular mobility at higher hydrocolloid concentration in the hydrocolloid-fruit ink decreased, resulting in the observed increased apparent viscosities (44).

The storage modulus (G′) and loss modulus (G^′′^), representing the elastic and viscous character of the samples were presented in Figures 1B, C, respectively. Generally, the G′ was higher than G^′′^ in all the samples: an indication of a higher elastic and solid-like than viscous character in the hydrocolloid-fruit inks. The hydrocolloids in the samples could have interacted and bound with components (e.g., water, starch, proteins) in pitaya fruit resulting in the increased solid-like character in the samples (45). Also, as the frequency increased, the values of G′ (Figure 1B) and G^′′^ (Figure 1C) increased in all samples, with generally higher values observed in CMC, then AG and XG containing hydrocolloid-fruit inks, especially at 9 g/100 g than 6 g/100 g hydrocolloid concentration. This was attributed to a high internal stiffness in samples, especially those prepared with CMC and AG at higher (9 g/100 g) hydrocolloid concentration due to network structures formed between hydrocolloid polymer chains (32). On the other hand, at lower (6 g/100 g) concentration and presence of XG in samples, the repelling nature of XG and incompatibility of surface charges between molecules in the mixtures could have resulted in a less solid character in the samples (32). As shown in Figure 1D, the loss tangent (tan δ, G^′′^/G′) values for all hydrocolloid-fruit inks were below 1. This implied a more elastic and solid-like character in the hydrocolloid-fruit inks. This was in agreement with studies that revealed that samples with low tan δ values exhibited more solid-like characters (46). Moreover, the tan δ values were observed to be lowest in XG, followed by AG (9 g/100 g < 6 g/100 g) and CMC (9 g/100 g < 6 g/100 g) containing hydrocolloid-fruit inks (Figure 1D). Changes in G^′′^ (Figure 1B) and G′ (Figure 1C) of the inks were disproportionately balanced, especially in the inks prepared with XG. The tan δ values were lower at higher (9 g/100 g) than lower (6 g/100 g) concentration of AG and CMC in the inks, an indication of increased elastic character at higher hydrocolloid concentration in the inks (46). This was attributed to the potential increase in complex networks formed at higher concentration of AG and CMC with the components in the pitaya fruit, resulting in restricted chain movement and decreased flexibility in the inks (47). Interestingly, tan δ values were not affected by the concentration of XG in inks. This was attributed to the proportional increase in G′ and G^′′^ in respect to change in XG concentrations in inks compared to AG and CMC in inks which resulted in the lower tan δ values observed (32). Higher elastic (solid-like) character of the 3D printing ink, the better the retention of the it's structure during and after the 3D printing process (31). Therefore, the dynamic oscillatory observations implied that the use of XG, followed by AG and CMC at higher concentration may provide better structural stability for 3D printing of pitaya fruit in the hydrocolloid-fruit ink.

### 3.4 Effect of type and concentration of hydrocolloid on the water mobility in hydrocolloid-fruit inks

**Figure 2:**
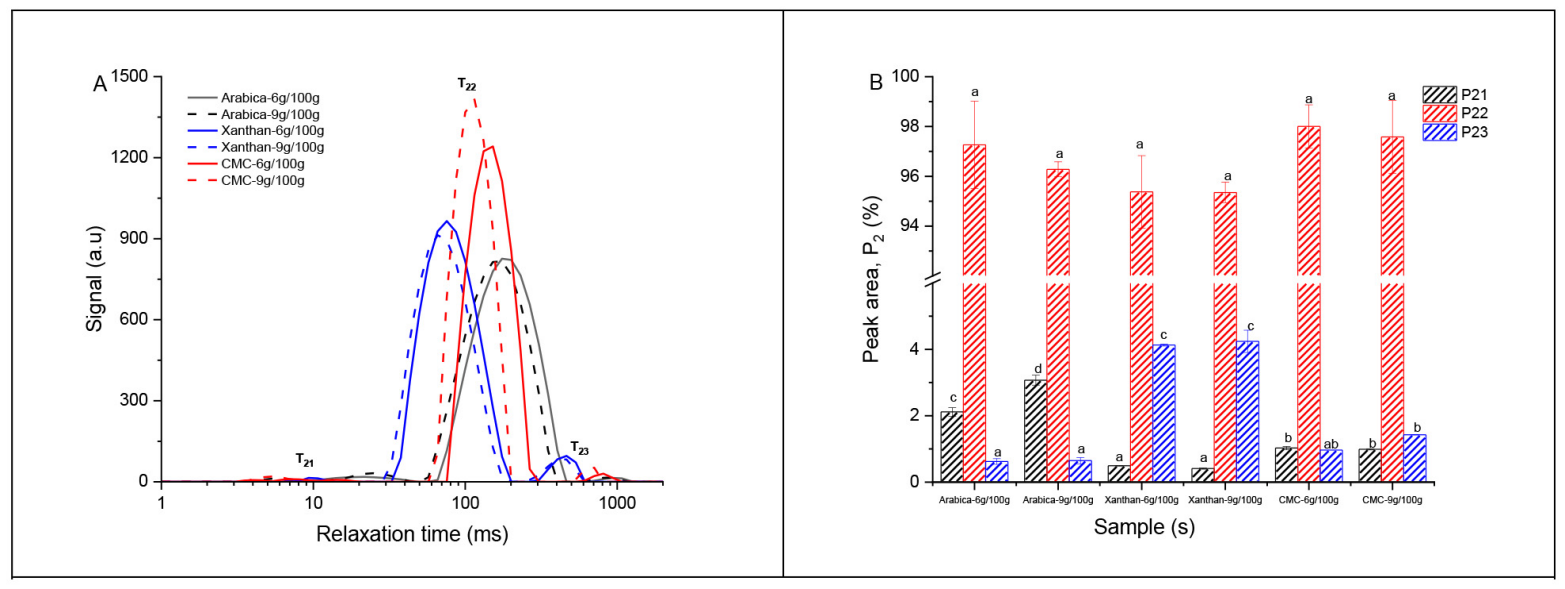
Effect of different hydrocolloids at two concentrations on the water mobility based on Carr-Purcell-Meiboom-Gill (CPMG) T_2_ curves **(A)** and T_2_ peak area percentage **(B)** of pitaya fruit-based inks. Means (*n* = 3) with different letters indicated significant differences at *p* < 0.05.

The transverse relaxation time (T_2_) and its distribution reflect different binding modes and dynamics of water mobility in complex food matrices (48). In this study, the changes in water mobility based on the transverse relaxation time (T_2_) and its distribution based on the relative peak area percentage distribution of the hydrocolloid-fruit inks were shown in Figure 2. As shown in Figure 2A, three relaxation peak populations (T_2_) of the samples were centered at 7.58–46.53 ms (T_21_), 174.75–403.7 ms (T_22_), and 351.12–1,232.84 ms (T_23_), representing bound water (T_21_), immobilized water (T_22_), and free water (T_23_), respectively (49). In all samples, the population area under T_22_ was the most dominant, followed by T_23_, and least in T_21_ (Figures 2A, B). The high population peak area under the immobilized water (T_22_, 95.35%−98.01%) (Figure 2B), especially, in samples prepared with CMC (97.58%−98.01%), followed by AG (96.28%−97.26%), and XG (95.35%−95.38%), suggested the effect of the different hydrocolloids and concentrations used on restricting water molecule mobility and its impact on the rheological properties of the hydrocolloid-fruit inks. In addition, the T_22_ peak relaxation time were shorter (shifted closer to 0 ms) at higher (9 g/100 g) than lower (6 g/100 g) hydrocolloid concentration in all the fruit inks (Figure 2A). This could be that at higher hydrocolloid concentration, the WHC increased and more water molecules were trapped, thereby, less water was mobile, resulting in a shortened relaxation time in the hydrocolloid-fruit inks (32). Based on the differences in chemical composition and structure of hydrocolloids used such as CMC are chemically modified cellulose derivatives with linear cellulose backbone substituted by carboxymethyl groups (32); XG are microbial-derived hydrocolloids with cellulose backbone of β-glucose-linked β-units substituted on glucose residues replaced by a side-chain trisaccharide such as mannose and glucuronic acid (50); and AG are plant-based hydrocolloids with a branched structure having a backbone composed of 1,3-linked β-D-galactopyranosyl units and the side chains comprised of two to five 1,3-linked β-D-galactopyranosyl units, joined to the main chain by 1,6-linkages (51), it affected the water mobility in the hydrocolloid-fruit inks through different mechanisms associated with the ability of the hydrocolloid to form intermolecular bonding with the starch and other components in the pitaya fruit (52). Therefore, the reduced water mobility and water molecule re-distribution due to increased WHC at higher concentration of hydrocolloids in the hydrocolloid-fruit ink resulted in water molecules slowly re-orienting themselves and remaining more held together by hydrogen bonds (53). Low water mobility in the inks could be desirable during 3D printing in retention of the 3D printed structure (31).

### 3.5 Effect of type and concentration of hydrocolloid on the texture profile of 3D printed hydrocolloid-fruit inks

**Figure 3:**
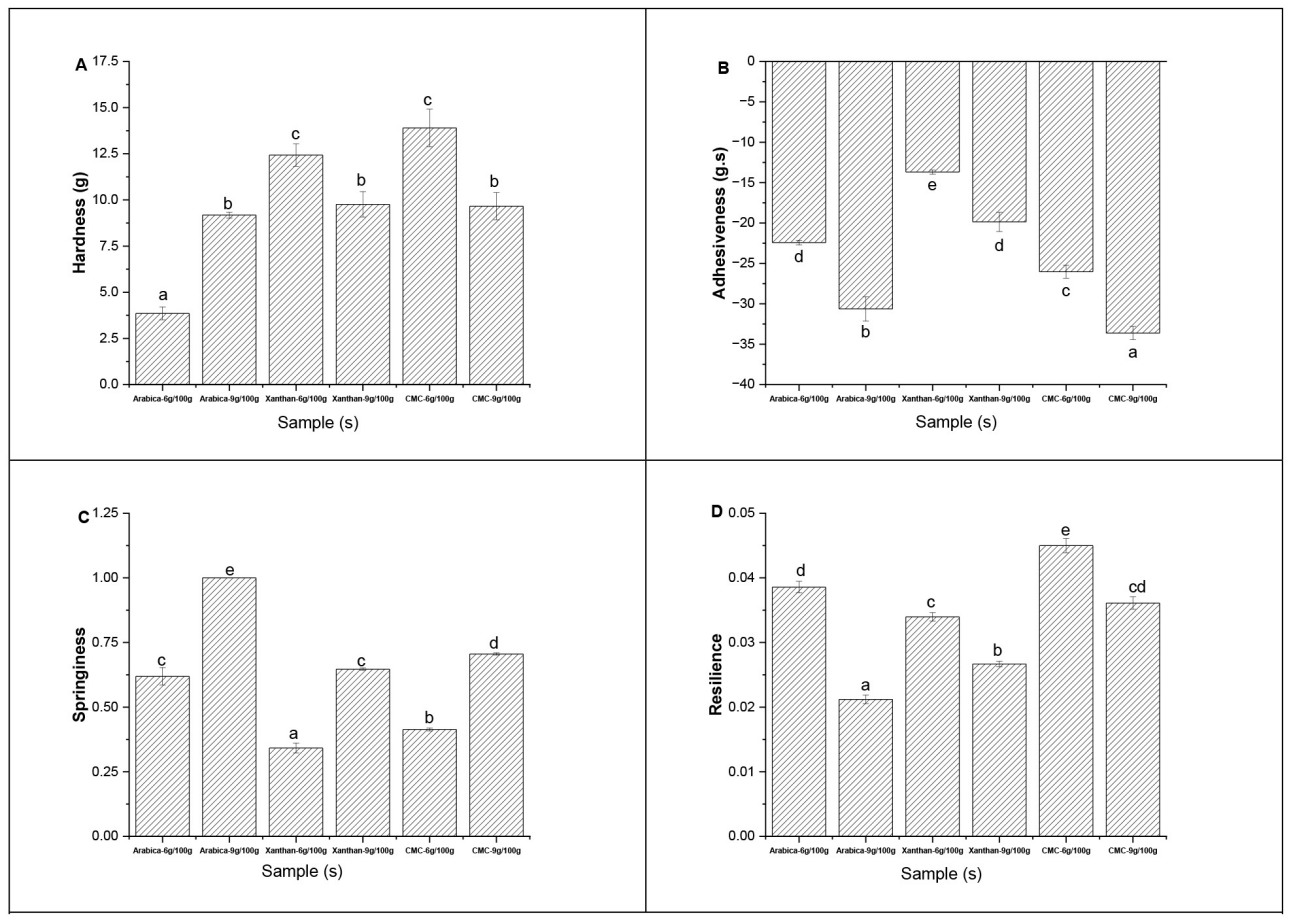
Effect of different hydrocolloids at two concentrations on the hardness **(A)**, adhesiveness **(B)**, springiness **(C)**, and resilience **(D)** texture profiles of different pitaya fruit-based inks. Means (*n* = 3) with different letters indicated significant differences at *p* < 0.05.

The textural profile of a food product is a vital property for its acceptance by consumers. The changes in the textural profile in terms of hardness, adhesiveness, springiness and resilience of the printed objects prepared with the different hydrocolloids at two concentrations were presented in Figure 3. Hardness (the maximum force needed to compress a food sample) was generally higher (*p* < 0.05) in printed objects prepared with CMC, followed by XG, and AG (Figure 3A). Moreover, hardness values were higher at 9 g/100 g than 6 g/100 g for CMC and XG in the printed objects. This was attributed to the high storage modulus (G′) (Figure 1B) and low tan δ (Figure 1D), leading to an increased mechanical strength in the respective hydrocolloid-fruit inks. The changes in hardness and G′ and tan δ were consistent with those reported by Feng et al. (22) for 3D printed β-carotene loaded yam starch-based hydrogel. Adhesiveness (the negative area after the first compression of sample) showed a downward trend, with much lower values observed in CMC, then XG, and AG at 9 g/100 g than 6 g/100 g in the printed object (Figure 3B). Foods with low adhesiveness value have been reported to easily form boluses suitable for safe swallowing and reducing the risk of chocking (8, 54). Hence, at higher concentration, the different hydrocolloids used in the printed objects were able to lubricate and reduce the friction in the 3D printed products. Springiness (the rate at which the sample returns to its former state before compression) was highest in AG, then CMC and XG printed objects, especially at 9 g/100 g than 6 g/100 g concentration (Figure 3C). On the other hand, the resilience (the ability of the sample to recover its original height) was highest in CMC, then XG and AG printed objects, especially at 6 g/100 g than 9 g/100 g (Figure 3D).

### 3.6 Effect of type and concentration of hydrocolloid on the color and printing properties of 3D printed hydrocolloid-fruit inks

The color properties of the 3D printed inks were represented by the L^*^ (lightness), a^*^ (redness), b^*^ (yellowness), were presented in Table 2. The type of hydrocolloid and concentration used in the 3D printed inks influenced the color profiles differently. For instance, the L^*^ (lightness) was highest in arabica (9 g/100 g than 6 g/100 g), while the a^*^ (redness) was highest in XG (6 g/100 g than 9 g/100 g), b^*^ (yellowness) was highest in CMC (9 g/100 g than 6 g/100 g) printed objects (Table 2). The changes in color may be associated with the interactions between the different hydrocolloids at different concentrations and other molecules such as starch and phenolic compounds in pitaya fruit to form complexes with varying particle sizes which altered the diffraction patterns (55).

**Table 2:** Effect of hydrocolloid type and concentration on the color properties and dimensional printing deviation of the printed objects.

**Sample (s)**	**3D printed ink**	**Color properties**			**3D printed ink dimension (mm)**			**Dimensional printing deviation (%)**		
		**L** ^*^	**a** ^*^	**b** ^*^	**L (mm)**	**W (mm)**	**H (mm)**	**L (mm)**	**W (mm)**	**H (mm)**
Arabica-6 g/100 g	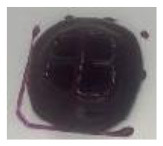	21.37 ± 1.77abc	12.74 ± 0.58c	1.16 ± 0.03b	30.00 ± 2.83bc	10.00 ± 1.41a	50.00 ± 7.07cd	50.00 ± 14.14bc	−16.67 ± 1179a	−16.67 ± 1179a
Arabica-9 g/100 g	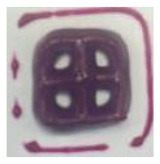	23.65 ± 1.27bc	9.60 ± 0.60b	0.63 ± 0.06a	27.00 ± 1.41bc	27.00 ± 0.00b	11.50 ± 2.12a	35.00 ± 7.07bc	35.00 ± 0.00b	−4.17 ± 17.68a
Xanthan-6 g/100 g	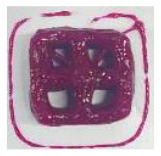	23.98 ± 1.82c	13.59 ± 1.84c	0.48 ± 0.12a	21.00 ± 1.41a	23.00 ± 1.41a	13.00 ± 1.41a	5.00 ± 7.07a	15.00 ± 7.07a	8.34 ± 11.79a
Xanthan-9 g/100 g	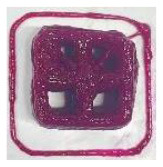	18.51 ± 1.13a	12.37 ± 1.55c	1.73 ± 0.15c	21.00 ± 1.41b	21.50 ± 0.71a	12.50 ± 0.71a	5.00 ± 7.07a	7.50 ± 3.54a	4.17 ± 5.89a
CMC-6 g/100 g	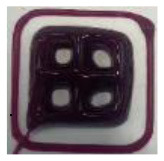	20.27 ± 1.52ab	6.64 ± 1.21a	1.36 ± 0.17b	31.00 ± 1.41d	31.00 ± 0.00c	11.00 ± 1.41a	55.00 ± 7.07d	55.00 ± 0.00c	−8.34 ± 11.79a
CMC-9 g/100 g	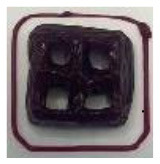	19.76 ± 3.16a	7.62 ± 0.75ab	1.78 ± 0.08c	24.00 ± 0.71ab	23.00 ± 1.41a	11.25 ± 1.06a	20.00 ± 3.54ab	15.00 ± 7.07a	−6.25 ± 8.84a

Mean (n = 3) with different lower-case letter in each column denotes difference (*p* < 0.05).

MC, Moisture content; WHC, Water holding capacity; CMC, Carboxymethylcellulose.

The effect of hydrocolloid type and the concentration on the printing precision (length, width, and height) and deviations in the 3D printed pitaya fruit inks were shown in Table 2. The higher the absolute value of the printed object, the larger the dimensional printing deviation and the lower the printing precision (22). The results in this study showed that all the dimensions of the printed objects increased. The highest increase in the length, width and height was generally seen in the printed objects containing AG, then CMC and least in XG. Moreover, a lower printing deviation was observed in the printed objects containing each hydrocolloid at 9 g/100 g than 6 g/100 g. Based on the rheological properties and water mobility observations, the changes in printing deviations were expected due to the higher solid-like (elastic) character and lower water mobility as the water holding capacity increased in the presence of XG, followed by CMC and AG at higher concentration (53). Subsequently, rheological and water mobility of the inks influenced the structure of the printed objects by improving the printing accuracy in the samples with the lowest dimensional printing deviation, including, XG (9 g/100 g < 6 g/100 g), then CMC (9 g/100 g < 6 g/100 g) and AG containing 3D printed pitaya ink objects (31).

### 3.7 Correlation analysis

**Figure 4:**
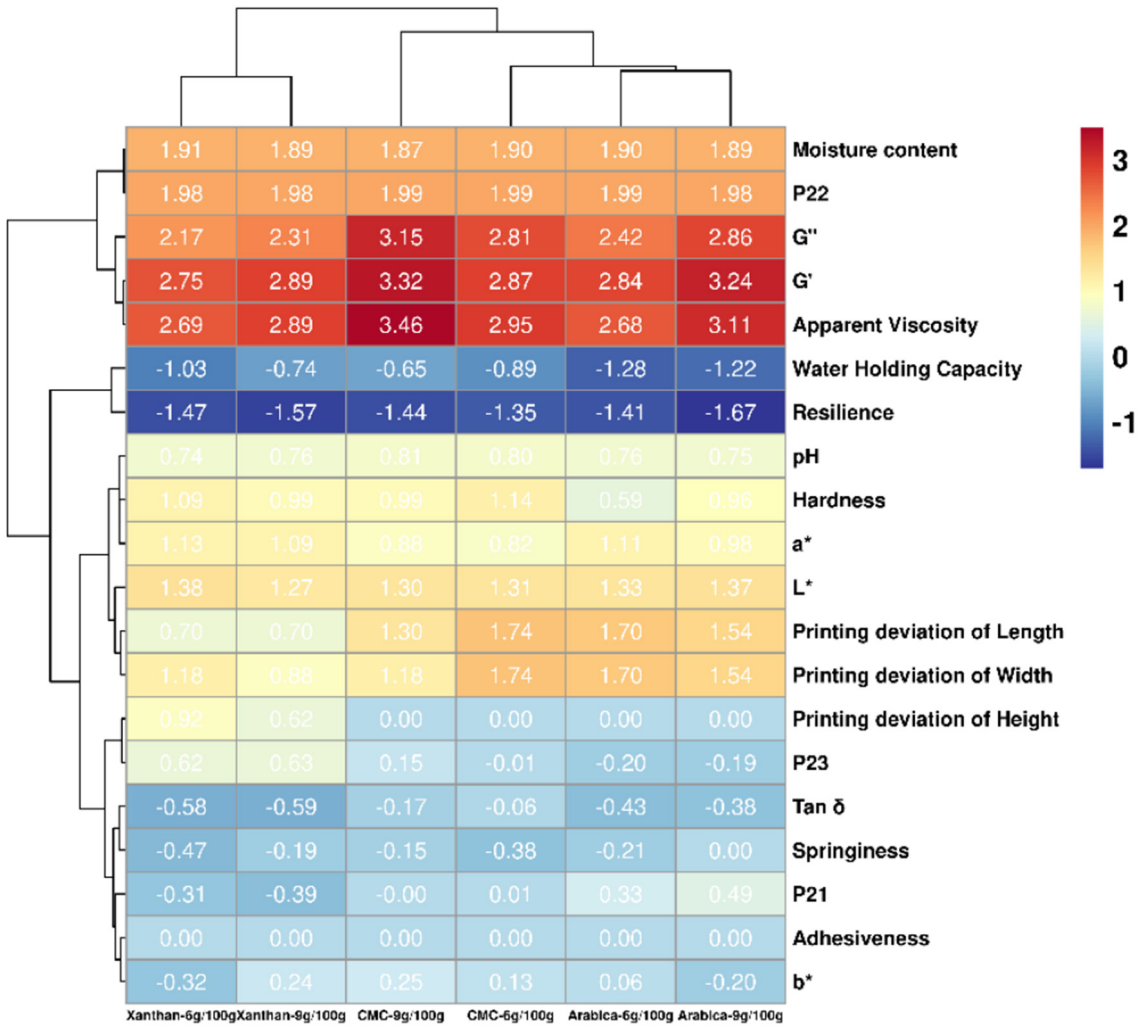
Hierarchical clustering heatmap analysis of the property parameters between samples prepared with different hydrocolloids at two concentrations presented as Euclidean distance. The areas shaded in red indicate a higher value, while the areas shaded in blue indicate a lower value.

Hierarchical cluster analysis was used to correlate the effect of the concentration and hydrocolloid type used on several parameters of the hydrocolloid-fruit inks and their printed objects. The results were presented in Figure 4. The average linkage and Euclidean distance showed that there were four main clusters of samples, the first cluster having samples prepared with XG (6 g/100 g and 9 g/100 g), the second cluster having CMC at 9 g/100 g concentration sample, the third cluster having CMC at 6 g/100 g concentration sample, and the fourth cluster having samples prepared with AG (6 g/100 g and 9 g/100 g) (Figure 4). On the other hand, six main clusters for the parameters were seen (Figure 4). The MC and P_22_ parameters were in the first cluster. The second cluster had the G^′′^, G′ and apparent viscosity parameters, with higher values seen in CMC-9 g/100 g, arabica-9 g/100 g, CMC-6 g/100 g, xanthan-9 g/100 g, arabica-6 g/100 g, and xanthan-6 g/100 g. The WHC and resilience were found in the third cluster, with higher values in CMC-9 g/100 g, xanthan-9 g/100 g, CMC-6 g/100 g, arabica-9 g/100 g, arabica-9 g/100 g, and xanthan-6 g/100 g. The fourth cluster included pH, hardness, a^*^, L^*^, printing deviation of the length and width. In this cluster, the hardness values were higher CMC-6 g/100 g, xanthan-6 g/100 g, xanthan-9 g/100 g, CMC-9 g/100 g, arabica-9 g/100 g, arabica-6 g/100g; while the printing deviation of length and width was lowest in xanthan-6 g/100 g and xanthan-9 g/100 g, but highest in CMC-6 g/100 g and arabica-6 g/100 g. The fifth cluster included the printing deviation of the height and P_23_, while the sixth cluster had tan δ, springiness, P_21_, adhesiveness and b^*^. These results implied that the textural profile and printing stability of the pitaya fruit inks were influenced by the type of hydrocolloid and concentration used which invariably impacted the physicochemical, rheological and water mobility of the inks. Subsequently, the hierarchical cluster analysis was a useful tool to classify the printing stability of pitaya fruit containing different hydrocolloids at different concentration.

## 4 Conclusion

This study explored the effects of three hydrocolloid types at two concentrations on improving the technological and functional properties of inks based on pitaya fruit and their 3D printed objects, including the water holding capacity, apparent viscosity, storage and loss moduli, water mobility, textural profile, color properties. and printing characteristics. Different types of hydrocolloids increased the water holding capacity, lowered the apparent viscosity, increased the pseudoplastic and solid-like character, and restricted the water mobility in the hydrocolloid-fruit inks. Moreover, better outcomes were observed in fruit inks prepared with XG, followed by CMC and AG hydrocolloids. With the exception of tan δ values of inks containing XG which were not affected by the XG concentration, higher (9 g/100 g) than lower (6 g/100 g) hydrocolloid concentration of XG, followed by CMC and AG in the hydrocolloid-fruit ink improved the printing precision, and significantly influenced the textural profile and color properties of the 3D printed hydrocolloid-fruit ink objects. These findings demonstrated the potential influence of hydrocolloid type at carefully selected concentrations on altering the physicochemical and technological properties of high moisture content fruit substrate-based inks for 3D food printing applications. Furthermore, these findings pave the way for further studies on the development of natural and health-promoting fruit substrate-based 3D printed snack food products.

## Data Availability

The original contributions presented in the study are included in the article/Supplementary material, further inquiries can be directed to the corresponding authors.
