# Supplementary material for: Effect of type and concentration of hydrocolloids on the rheology, water mobility, and 3D printing properties of pitaya fruit-based ink
Source: Front Nutr. 2026 Jan 6;12:1668661. doi: 10.3389/fnut.2025.1668661 (PMC12815879; doi:10.3389/fnut.2025.1668661)
Supplement: Supplementary file 1 [file Image_1.pdf]

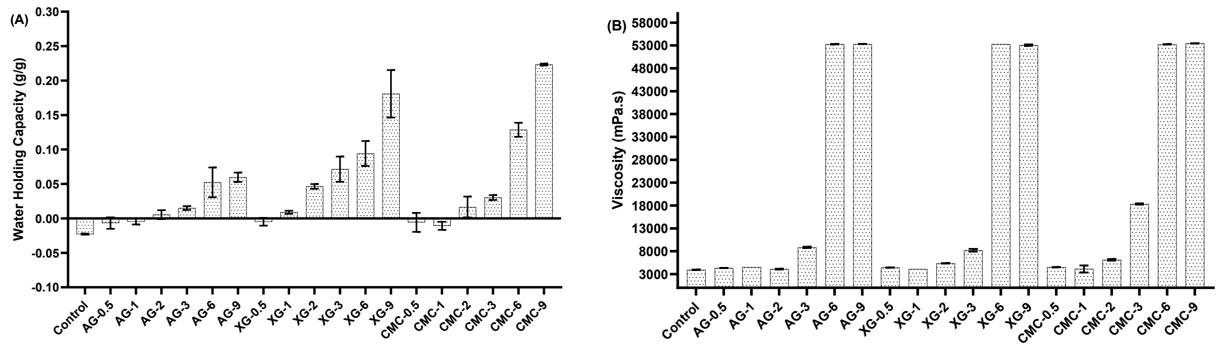


Supplementary Figure S1. Technological properties, water holding capacity (A) and viscosity (B) of hydrocolloid-fruit inks. Where, AG: Arabica gum, XG: Xanthan gum, CMC: Carboxymethylcellulose, at concentration 0.5g/100g (-0.5), 1g/100g (-1), 3g/100g (-3), 6g/100g (-6) and 9g/100g (-9), respectively.
